# Acute single channel EEG predictors of cognitive function after stroke

**DOI:** 10.1371/journal.pone.0185841

**Published:** 2017-10-02

**Authors:** Anna Aminov, Jeffrey M. Rogers, Stuart J. Johnstone, Sandy Middleton, Peter H. Wilson

**Affiliations:** 1 School of Psychology, Australian Catholic University, Sydney, NSW, Australia; 2 South Eastern Sydney Local Health District, Sydney, NSW, Australia; 3 School of Psychology, University of Wollongong, Wollongong, NSW Australia; 4 Nursing Research Institute, St Vincent’s Health Australia and Australian Catholic University, Sydney, NSW Australia; 5 School of Psychology, Australian Catholic University, Melbourne, VIC, Australia; 6 Centre for Disability and Development Research, Australian Catholic University, Melbourne, VIC, Australia; Wadsworth Center, UNITED STATES

## Abstract

**Background:**

Early and accurate identification of factors that predict post-stroke cognitive outcome is important to set realistic targets for rehabilitation and to guide patients and their families accordingly. However, behavioral measures of cognition are difficult to obtain in the acute phase of recovery due to clinical factors (e.g. fatigue) and functional barriers (e.g. language deficits). The aim of the current study was to test whether single channel wireless EEG data obtained acutely following stroke could predict longer-term cognitive function.

**Methods:**

Resting state Relative Power (RP) of delta, theta, alpha, beta, delta/alpha ratio (DAR), and delta/theta ratio (DTR) were obtained from a single electrode over FP1 in 24 participants within 72 hours of a first-ever stroke. The Montreal Cognitive Assessment (MoCA) was administered at 90-days post-stroke. Correlation and regression analyses were completed to identify relationships between 90-day cognitive function and electrophysiological data, neurological status, and demographic characteristics at admission.

**Results:**

Four acute qEEG indices demonstrated *moderate* to *high* correlations with 90-day MoCA scores: DTR (*r* = -0.57, *p* = 0.01), RP theta (*r* = 0.50, *p* = 0.01), RP delta (*r* = -0.47, *p* = 0.02), and DAR (*r* = -0.45, *p* = 0.03). Acute DTR (*b* = -0.36, *p* < 0.05) and stroke severity on admission (*b* = -0.63, *p* < 0.01) were the best linear combination of predictors of MoCA scores 90-days post-stroke, accounting for 75% of variance.

**Conclusions:**

Data generated by a single pre-frontal electrode support the prognostic value of acute DAR, and identify DTR as a potential marker of post-stroke cognitive outcome. Use of single channel recording in an acute clinical setting may provide an efficient and valid predictor of cognitive function after stroke.

## Introduction

Cognitive impairment is a common and persistent sequela of stroke [1–3] and a major contributor to long-term disability [4], poorer functional recovery [2, 5], and reduced quality of life [6, 7]. Moreover, cognitive impairment can diminish the efficacy of rehabilitation interventions [8] and significantly elevate the risk for psychological problems such as depression and anxiety [9]. Not surprisingly, the cost of stroke care in patients with cognitive impairment is three times higher than those without [1, 4]. Early and accurate identification of factors that predict cognitive outcomes are needed to inform clinical decisions about required levels of care, to set realistic targets and strategies for rehabilitation, and to guide patients and their families accordingly [8, 10, 11].

Nevertheless, patients routinely fail to receive any “baseline” cognitive evaluation until months after their stroke due to clinical factors (e.g. fluctuating levels of arousal, distress, confusion, headache, fatigue) and functional barriers (e.g. sensory, language, motor deficits) that limit an individual’s ability to complete cognitive testing [10, 11]. These issues are only exacerbated in the acute setting [4, 8]. Stroke survivors with “unfavourable neurological outcomes” are therefore often excluded from any acute cognitive assessment, and may not undergo cognitive testing for several weeks to months after injury [8, 11].

Quantitative electroencephalography (qEEG) has long been utilized in clinical practice to detect, describe, and monitor brain function in both healthy individuals and following neuro-trauma [12]. Due to its non-invasive nature, qEEG is regarded as a highly effective addition to the traditional clinical evaluation in patients presenting with an altered or “difficult-to-assess mental status” [13, 14]. Furthermore, the feasibility of obtaining resting state qEEG data during the acute phase of brain injury has been demonstrated repeatedly, particularly in stroke [14–16]. Results from acute ischemic and hemorrhagic stroke populations have consistently identified the superiority of delta, and delta-derived qEEG metrics such as the delta/alpha ratio (DAR) over traditional measures of stroke severity in predicting longer-term functional outcomes [16, 17].

More recently, acute DAR obtained at rest was also shown to correlate with cognitive outcome following stroke [18]. From the 18-electrode array applied, the strength of relationship was greatest at pre-frontal electrodes, encouraging the future use of a more localised recording paradigm [18]. However, cognitive outcome was assessed using the subjective clinician report Functional Independence Measure and Functional Assessment Measure [FIM-FAM; 19]. Standardised psychometric testing is the preferred method to determine cognitive status and measure impairment [20, 21]. Rating scales often lack specific assessment tasks to elicit relevant behaviours, and in particular, poor reliability has been reported for the cognitive items of the FIM-FAM due to the ambiguity examiners encounter attempting to operationalize these components of the instrument [22]. In addition, the large variability in the timing of FIM-FAM completion (range 79–209 days) obscured the nature of the intended 90-day outcomes evaluation. Finally, EEG studies to date have not made use of normative data, which could allow comparison of the outcomes of stroke patients with a healthy population, to better describe the degree of any observed abnormalities.

Advances in EEG technology now provide wireless single-channel frontal electrode systems that seek to improve usability and portability [23–25], while maintaining data quality [26, 27]. The ease and efficiency of data collection with these portable devices is well suited to the clinical environment, where collection via conventional lab-based, multi-electrode montages can be cumbersome [26, 27] and redundant [18]. The aim of the current study was to progress previous work by focusing only on a single pre-frontal electrode at the time of qEEG data collection, and administering an objective, standardised cognitive assessment tool at a strict 90-day follow-up. Compared to healthy older adult norms, it was predicted that stroke survivors would demonstrate acute single-channel qEEG abnormalities, which were expected to correlate with 90-day cognitive function.

## Methods

### Participants

From August 2014 to October 2015 participants were consecutively recruited by AA from the acute stroke unit of a major tertiary hospital in Sydney, Australia. Patients admitted to the unit within 72 hours of a first-ever stroke were eligible for participation. Time of stroke onset was defined as the time the participant was last seen without stroke symptoms, as documented in the medical records. Individuals with a previous history of neurological or psychiatric disorder, non-English speaking, or under 18 years of age, were excluded. Stroke nurses assisted AA in identification of eligible candidates.

### Outcome measures

Cognitive function was assessed using the Montreal Cognitive Assessment [MoCA; 28]. This 12-item screening instrument surveys cognitive function across the domains of orientation, attention, language, visuospatial, memory, and executive function [4, 29]. The MoCA has demonstrated validity in stroke [4, 28, 29], and is widely used to assess outcomes following acquired brain injury [4]. The MoCA is scored out of 30 with scores below 26 suggestive of cognitive impairment [4].

### EEG data acquisition and analysis

Continuous EEG was collected during a single 3-min eyes closed resting state condition using the NeuroSky Mindset device (NeuroSky^TM^, USA). The device acquires continuous EEG from a single dry stainless steel electrode positioned at the International 10–20 system site FP1. Signals were sampled continuously at a rate of 128 Hz. Raw EEG data was transmitted wirelessly by Bluetooth to a laptop computer for recording and subsequent off-line quantitative analysis.

SCAN Edit version 3 software (Neuroscan^TM^, USA) was used to analyse EEG data. The raw EEG waveform data was amplified with a Neuroscan Inc. (Herndon, VA) SynAmps system with a band-pass filter of 0.5–30 Hz, and manually inspected to identify any movement or muscle artifact. Identified sections were marked and excluded from further processing. Remaining epochs containing amplitudes in excess of ±100μV were removed using the rejection filter included in the SCAN software. Artifact-free 4-sec EEG epochs (1/4 Hz resolution) were submitted to Fast Fourier Transforms (FFT), with 10% Hamming to extract the absolute power in the following frequency bands: delta (1.5–3.5 Hz), theta (3.5–7.5 Hz), alpha (7.5–12.5 Hz), and beta (12.5–25 Hz). Relative power (RP) was calculated by summing absolute power across the four bands to compute total power, and then dividing the absolute power for each individual band by the total power, expressed as a percentage. The delta/alpha ratio (DAR) and the delta/theta ratio (DTR) were computed by dividing the RPs of the relevant frequency bands.

### Procedure

After obtaining informed consent, the National Institute of Health Stroke Scale (NIHSS; [30]) and Modified Rankin Scale [mRS; 31] were completed at hospital bedside within 72 hours of stroke symptom onset to document the extent of stroke-related neurological deficits and global disability, respectively. With both instruments, higher scores reflect poorer outcome. The Oxfordshire Community Stroke Project Classification [32] was used to classify stroke into four categories: Total Anterior Circulation Stroke, Partial Anterior Circulation Stroke, Posterior Circulation Stroke and Lacunar Stroke. A family member of the patient completed the Short Form of the Informant Questionnaire on Cognitive Decline in the Elderly (Short-IQCODE), to rule out the presence of pre-morbid dementia [33].

Continuous EEG was obtained at hospital bedside within 72 hours of stroke symptom onset. The single electrode was placed at FP1 according to the International 10–20 system, using 10% divisions of the inion-to-nasion distance. After minimising signal impedance, participants were asked to close their eyes and relax for the 3-min recording session. At 90-days post-stroke, participants were administered the MoCA at a location of their convenience. All study instruments were administered by AA following specialist training with experienced clinicians. For their participation, patients received $10 gift vouchers. This research was approved by Australian Catholic University and South Eastern Sydney Local Health District Human Research Ethics Committees, and each participant (or their carer/substitute decision maker) provided written informed consent for voluntary participation.

Previous research examining the effectiveness of DAR in predicting 90-day cognitive functioning in stroke patients [18] reported a significant correlation equivalent to a *large* effect size (d = 0.66; [34]). Sample size calculations in the current study were based on the conservative assumption that the single channel EEG device might produce a smaller effect. Power calculations computed using G*Power 3.1.7 [35] identified that with a one-sided alpha level of 0.05 and power of 85%, the current planned correlational analysis required a total sample size of 19 participants to detect a relationship equating to a *large* (*d* = 0.55) effect.

### Statistical analysis

Grand averaged RP values were compared with published age-based normative data [27] using independent samples *t*-tests. Pearson product-moment correlations were computed between 90-day MoCA Total Scores and neurophysiological (i.e. delta, theta, alpha, beta, DAR, DTR), neurological (i.e. NIHSS, mRS,) and demographic (i.e. age, years of education) data collected within the first 72 hours after admission. Statistically significant variables were entered into hierarchical linear regression analyses to explore the combination of neurological measures, demographic characteristics, and neurophysiological data that best predicted 90-day cognitive outcomes. All analyses were completed with IBM SPSS Statistics for Windows, version 23 (IBM Corp., Armonk, N.Y).

## Results

Twenty-four participants were initially recruited into the study. Over the intervening 90-days between EEG acquisition and cognitive testing two participants passed away, and three declined the follow-up. Complete data were subsequently available for 19 participants (Table 1). There were no significant differences in the admission characteristics of participants who did not complete data collection and the final study cohort (Table 2). All participants were right-handed and had sustained first-episode stroke, confirmed on neuroimaging as part of the routine assessments provided by their treating team. No participant scored above the clinical cut-off score of 4 on the Short-IQCODE [33]. In general, the stroke severity and disability of participants was *mild* as classified by the NIHSS and mRS.

**Table 1:** Demographic, neurological, and cognitive characteristics of participants.

Age^a^	65.95 (15.78), 33–93
Gender^b^	
Male	12 (63)
Female	7 (36)
Education Level^b^	
< 12 years	7 (37)
12 years	4 (21)
> 12 years	8 (42)
Baseline NIHSS score^a^	5.74 (6.23), 0–18
Mild (< 8)^b^	13 (68)
Moderate (8–15)^b^	3 (16)
Moderately severe (16–20)^b^	3 (16)
Baseline mRS score^a^	1.86 (1.36), 0–4
0 ^b^	2 (10)
1 ^b^	9 (47)
2 ^b^	1 (5)
3 ^b^	4 (21)
4 ^b^	3 (15)
Ischemic Stroke^b^	15 (79)
Hemorrhagic Stroke^b^	4 (21)
Left-sided lesion^b^	8 (42)
Right-sided lesion^b^	11 (58)
Oxfordshire stroke classification	
TACI/H^b^	1 (5.27) 2 (10.53)
PACI/H^b^	5 (26.32) 0
POCI/H^b^	5 (26.32) 0
LACI/H^b^	4 (21.05) 2 (10.53)
Time to EEG recording (hours)^a^	46.68 (18.84), 20–72
90 day follow-up (days)^a^	95.21 (7.68), 80–111
MoCA total score^a^	21.57 (4.64), 12–29

^a^Mean (SD) range

^b^No (%).

Note: LACI/H, lacunar infarct/ hemorrhage; mRS, Modified Rankin Scale, range 0–6; NIHSS, National Institute of Health Stroke Scale range 0–24; PACI/H, partial anterior circulation infarct/ hemorrhage; POCI/H, posterior circulation infarct/ hemorrhage; TACI/H, total anterior circulation infarct/ hemorrhage.

**Table 2:** Demographic and neurological characteristics of the final cohort (n = 19) and drop-outs (n = 5).

	Final Cohort^a^	Drop-outs^a^	Significance testing
Age	65.95 (15.78)	76.67 (6.03)	*t*(1) = 1.14, *p* = 0.23
Years of education	13.00 (3.74)	9.00 (1.00)	*t*(1) = 1.81, *p* = 0.09
NIHSS	5.73 (6.23)	4.00 (3.61)	*t*(1) = 0.46, *p* = 0.65
Baseline mRS	1.84 (1.34)	2.00 (1.73)	*t*(1) = -0.18, *p* = 0.86
Admission recording interval (hours)	46.68 (18.84)	34.00 (28.58)	*t*(1) = 1.02, *p* = 0.32

^a^Mean (SD).

Note: mRS, Modified Rankin Scale, range 0–6; NIHSS, National Institute of Health Stroke Scale range 0–24.

**Fig 1:**
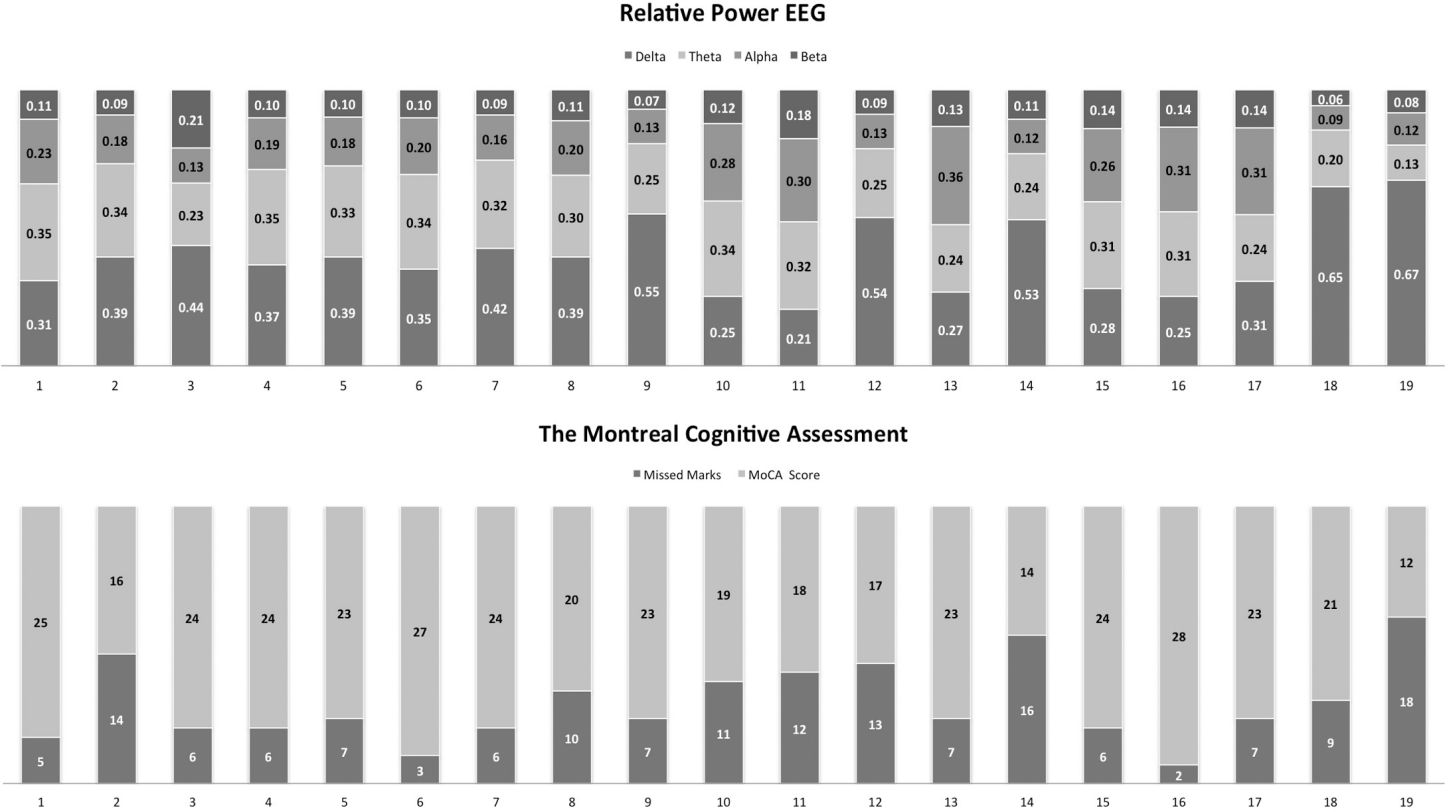
Individual participant. a) RP values for each of the four frequency bands collected at baseline b) MoCA cognitive outcomes at 90-days (Max Total Score = 30).

EEG was collected on average within the first two days after stroke (Table 1). On average, 20 valid qEEG epochs were obtained per participant, above the minimum threshold for analysis [26]. Mean RP for the delta, theta, alpha and beta frequencies are presented in Fig 1. Results from the stroke participants were compared to healthy older adult normative data [27], matched for gender [63% male c.f. 47% male; χ^2^(1) = 0.33], years of age [65.95 c.f. 64.84; *F*(1,36) = 0.07, *p* = 0.79], and years of education [13.00 c.f. 12.95; *F*(1,36) = 0.01, *p* = 0.96]. In stroke participants, the proportion of delta was significantly higher than older adult norms (Table 3). As well, there was significantly less theta and alpha activity in stroke participants. Accordingly, DAR values were significantly higher in stroke participants than the normative group. DTR values were similarly elevated. Participants were followed up on average 95 days after stroke (range 80–111 days). At this time 16 participants (84%) exhibited cognitive impairment [total score <26; [28]] on the MoCA (Table 1 and Fig 1).

**Table 3:** qEEG results for the stroke participants (n = 19) and the normative sample (n = 19; [27]).

	Stroke Group^a^	Normative Group^a^	Significance Testing
RP delta	0.39 (0.13)	0.28 (0.07)	*t*(26.65) = 3.28, *p* < 0.01, *d* = 1.07
RP theta	0.28 (0.06)	0.33 (0.05)	*t*(36) = -2.56, *p* = 0.02, *d* = 0.83
RP alpha	0.20 (0.08)	0.25 (0.07)	*t*(36) = -2.02, *p* = 0.05, *d* = 0.66
RP beta	0.11 (0.04)	0.13 (0.04)	*t*(36) = 1.33, *p* = 0.44, *d* = 0.50
DAR	2.57 (1.88)	1.26 (0.59)	*t*(21.46) = -2.88, *p* < 0.01, *d* = 0.93
DTR	1.60 (1.11)	0.87 (0.21)	*t*(19.29) = -2.83, *p* = 0.01, *d* = 0.92

^a^Mean (SD).

Note: DAR: delta/alpha ratio; DTR: delta/theta ratio; RP: relative power.

Correlations between 90-day MoCA Total Scores and neurophysiological, neurological and demographic variables on admission are summarised in Table 4. Four qEEG indices demonstrated significant correlations with MoCA results. Greater RP of theta was associated with better cognitive outcomes, while greater values of delta, DTR, and DAR were associated with poorer cognitive outcomes. Increasing age, stroke severity, and post-stroke disability were also significantly associated with greater cognitive impairment at 90-days, while further years of education was related to better MoCA cognitive outcomes.

**Table 4:** Correlations between 90-day MoCA scores and acute qEEG metrics, neurological measures, and demographic characteristics for all participants (n = 19) and only participants with ischemic stroke (n = 15).

	MoCA (All Strokes)	MoCA (Ischemic Strokes)
RP delta	-0.47*	-0.65**
RP theta	0.50*	0.66**
RP alpha	0.33	0.42
RP beta	0.18	0.28
DAR	-0.45*	-0.57*
DTR	-0.57**	-0.71**
NIHSS	-0.74**	-0.74**
MRS	-0.67**	-0.62**
Years of Education	0.40*	0.50*
Age	-0.51*	-0.61*

** Correlation is significant at the 0.01 level (1-tailed).

*Correlation is significant at the 0.05 level (1-tailed).

DAR: delta/alpha Ratio; DTR: delta/theta Ratio; MRS: Modified Rankin Scale: NIHSS, National Institute of Health Stroke Scale; RP: Relative Power.

**Fig 2:**
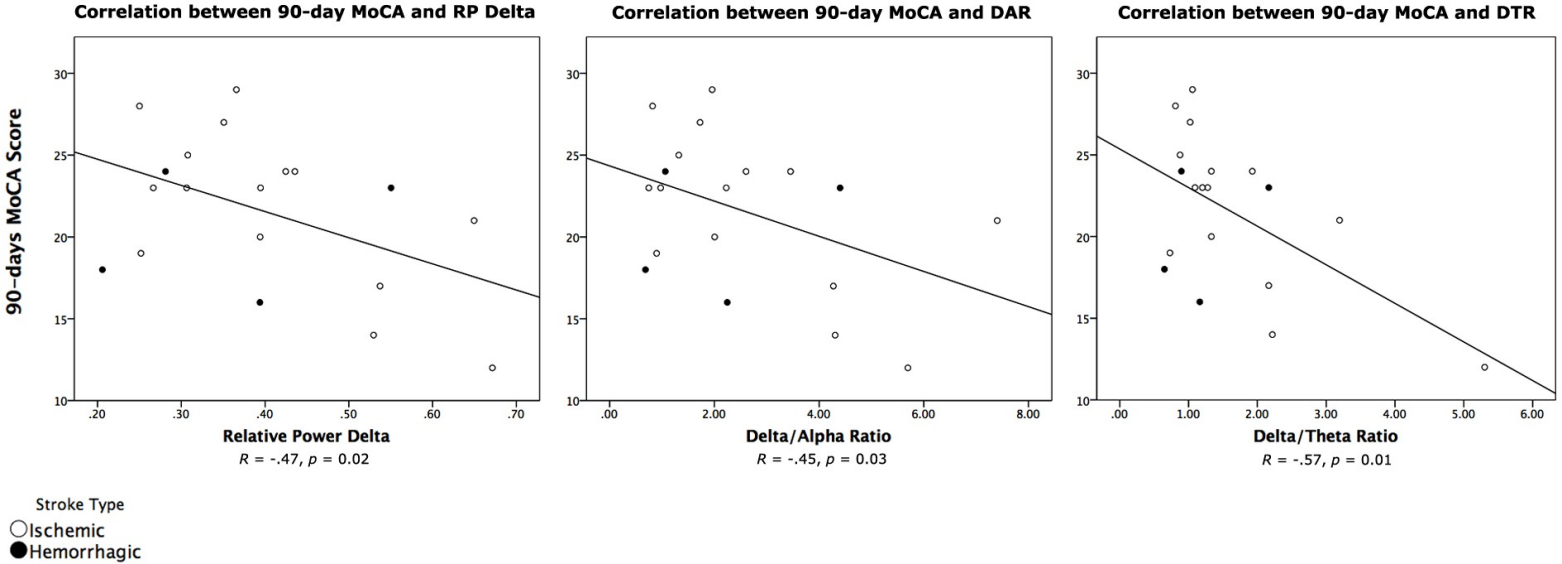
Individual correlations between acute qEEG indices and 90-day MoCA Total Score by stroke type.

Hemorrhagic stroke populations have been studied separately to ischemic populations, as distinct cognitive profiles [36, 37], including more severe cognitive impairment [36], have been reported in hemorrhagic stroke. In the current study, analyzing patients with ischemic stroke separately (*n* = 15), all correlations remained significant and were in fact slightly higher relative to those for the total sample (*n* = 19; Table 4). With no evidence hemorrhagic stroke participants were creating skew in the data, we retained all participants in further analyses. For reference purposes, however, the two clinical subgroups were identified using unique markers in scatterplots of key correlations (Fig 2).

For regression analysis, previously documented predictors (NIHSS, age, years of education) of neurological deficits in stroke [8, 10, 11] were added in the first block (stepwise method; Table 5). Statistically significant qEEG indexes (delta, theta, DAR, DTR) were added in the second block (stepwise method). The final model containing DTR and NIHSS score on admission significantly predicted 90-day MoCA outcome scores [*F*(4, 15) = 5.50, *p* < 0.01, *AdjR*^*2*^ = 0.70], accounting for 75% of the variance. Age and years of education did not contribute significant unique variance in the final model.

**Table 5:** Stepwise linear regression analysis of 90-day cognitive outcomes.

	B	SE B	Standardised *β*	95% CI for Odds Ratio	R (part)
Step 1					
Constant	30.84	2.93			
NIHSS	-0.483	0.114	-0.65**	[-0.72, -0.24]	-0.62
Age	-0.098	0.045	-0.33*	[-0.19, -0.03]	-0.32
Step 2					
Constant	29.64	2.64			
NIHSS	-0.47	0.101	-0.63**	[-0.68, -0.25]	-0.60
Age	-0.04	0.05	-0.15	[-0.14, 0.05]	-0.13
DTR	-1.50	0.64	-0.36*	[-2.86, -0.14]	-0.30

Note: *R*^2^ = 0.65 for step 1: *R*^2^ = 0.75 for step 2 (*p* < 0.001).

*p < 0.05.

**p < 0.01.

Variables excluded from step 1: Years of Education; step 2: RP Delta, Theta, and Delta/Alpha Ratio.

## Discussion

Stroke is associated with immediate brain changes including a biochemical cascade that can ultimately lead to cell death and cerebral infarction [15, 37, 38]. EEG may be sensitive to the effects of these acute changes [13], as it measures voltage oscillations resulting from the toxic production of lactic acid [39] and free radicals [40], calcium accumulation [40], protein degeneration [40], and loss of transmembrane gradients [15]. Monitoring these neurophysiological correlates of stroke neuro-trauma with a conventional multi-electrode EEG montage has previously been shown to correlate with clinician-rated impressions of longer-term cognitive function [18]. In the current study, we explored the capability of a single channel wireless EEG device to reproduce this prognostic relationship. To address shortcomings of previous research the current study also utilized a standardised measure of cognitive function, maintained a strict follow-up time frame, and compared EEG results against age-, gender-, and education-matched normative data.

Relative band-power of the delta, theta, and alpha EEG frequencies, and resulting DAR and DTR ratio measures of the relative intensity of abnormal slow-wave activity, were statistically significantly disturbed in the first 72 hours after stroke, compared with normative older adult data [27]. The qEEG results derived in the current study from a single pre-frontal recording channel were also in keeping with the typical profile of post-stroke electrophysiological abnormalities obtained from conventional lab-based systems [14, 15, 17, 41].

Specifically, excessive delta power is typically detectable at pre-frontal electrodes after stroke [12, 13, 17]. The proportion of RP delta activity was significantly higher in our stroke patients than normative older adult data [27], and was significantly related to 90-day cognitive functioning on the MoCA. The proportion of RP alpha activity was significantly lower in stroke participants than normative older adult data. However, RP alpha was not a significant predictor of cognitive function in the current study, suggesting it is less informative than other qEEG metrics for acute assessment and monitoring [17]. Beta activity was equivalent between stroke participants and normative older adult data, and demonstrated no relationship with longer-term cognitive function. Beta is considered the least reliable qEEG index [17, 41] and to date there have been no reported correlations between beta activity and cognitive or functional outcomes after stroke [17, 41].

Theta activity has been criticised as an unreliable measure of post-stroke pathophysiology, in part because estimates of RP in this frequency can be confounded by slowed alpha activity [17]. However, theta activity appears sensitive to the effects of post-stroke pathophysiology [13] and capable of discriminating between stroke patients and healthy controls [42], and of predicting functional outcomes [14] and cognitive impairment [43, 44]. In the current study, stroke patients demonstrated significantly attenuated theta activity compared with normative older adult data [27], and this frequency band was significantly correlated with cognitive functioning at 90-days. While to our knowledge theta activity has not previously been utilized to predict post-stroke cognitive deficits, greater levels of prefrontal theta activity have been identified as an indicator of healthy aging and superior cognitive function in older adults [45], while decreased frontal theta is related to decline in working memory [46, 47] and memory function [48, 49] in older adults.

DAR has most often been identified as the EEG metric possessing the greatest utility in predicting post-stroke outcomes [17], including enduring neurological symptoms [50], clinician rated impressions of functional disability [7, 18], as well as clinician rated impressions of cognitive impairment [18]. In the current study, DAR calculated from a single pre-frontal electrode was significantly elevated relative to normative older adult data [27]. DAR also significantly correlated with 90-day cognitive functioning, but to a lesser extent than previously reported [-0.45 c.f. -0.66; 18]. This is likely to be due to the increased specificity of an objective and standardised measure of cognitive function [20, 21] used in the current study, as well as a minor trade-off in terms of data quality associated with the use of a single-channel EEG system [26].

The most significant acute electrophysiological marker of longer-term cognitive functioning in the current study was DTR. DTR has not previously been identified as a potential predictor of either functional or cognitive outcomes after stroke, although it is unknown whether this may be due to a lack of investigation of this qEEG ratio, or a previous lack of statistically significant findings in relation to this metric (i.e. a file drawer effect). Abnormal delta index post-stroke may influence attentional capacity [12, 51, 52], which appears to be a key determinant of functional and cognitive outcomes [14, 16, 17]. Abnormal theta activity post-stroke may be related to delayed verbal recall [53, 54], reduced language processing and comprehension [54, 55], as well as problems with attention [44, 48], memory [43, 44] and executive function [45]. A metric combining delta and theta, the two most statistically significant individual EEG frequencies in the current study, would appear to optimise the prognostic capabilities of acute electrophysiological data, and the current findings encourage further research on the value of DTR in future evaluations of post-stroke outcomes.

Regression analyses confirmed the prognostic value of DTR, revealing the qEEG metric uniquely explained 19% of the variability in 90-day post-stroke cognitive outcome. Other qEEG frequencies and ratios were not statistically significant predictors. Previously reported demographic variables such as age and education [8, 10, 11] were also not significant predictors in the current model. NIHSS is a widely used measure of stroke severity [56, 57], and acute ratings correlate with cognitive outcome after stroke [57, 58]. In the current regression model, acute NIHSS scores uniquely explained 56% of the variance in longer-term cognitive outcome.

Taken as a whole, acute clinical ratings of neurological status (i.e. NIHSS) combined with pre-frontal neurophysiological measurement explained 75% of the variance in MoCA scores 90-days post-stroke, appearing to provide a powerful acute model of longer-term cognitive function. Furthermore, single channel qEEG monitoring is not hampered by the clinical factors and functional barriers that frequently confound standard behavioural assessments [11, 14, 15], which may ease the process of acute identification of cognitive status, and subsequently enhance clinical evaluations, treatment planning, and goal setting following stroke.

Functional biomarkers obtained via magnetic resonance imaging (MRI) techniques may also offer prognostic value in identifying post-stroke cognitive impairment, and a combined study of EEG and MRI would enable observation of brain network dynamics with high spatio-temporal resolution. Using resting-state functional MRI (rs-fMRI), disruptions to network connectivity have been demonstrated within the first two weeks after stroke, which may represent diminished processing capacity [59, 60]. Resting state fMRI in stroke patients 3–14 days after injury has also been used to identify regions of hypo-perfusion [61], but these radiological findings are yet to be examined in relation to cognitive outcome measures. Finally, utilising structural imaging, the degree and distribution of whole-brain, deep, and cholinergic white matter lesions detected within one-week after stroke has previously been associated with cognitive impairment [62–64]. However, despite these promising findings, a portable and easily administered single channel EEG device can offer time and cost-effectiveness in acute settings not currently achievable using either MRI or multi-channel EEG system technologies [13, 26, 65, 66].

Investigations of stroke outcomes often utilise mixed populations [7, 15] and the current study also combined data from both ischemic and hemorrhagic stroke survivors. While some researchers have preferred to analyse these populations separately, the difference between ischemic and hemorrhagic strokes might not be as great in terms of cognitive outcomes [5, 29], and qEEG sensitivity to these outcomes [7, 15]. Ultimately, both ischemic and hemorrhagic stroke conditions lead to cerebral infarction, and delta and theta related qEEG metrics may detect the biochemical changes associated with a brain moving into that state [15, 38]. Therefore, delta and theta related qEEG data may be sensitive to all forms of stroke, and not specific to the type of stroke. Consequently, it appears that when delta is elevated and theta suppressed acutely, a poorer longer-term cognitive prognosis can be expected, regardless of stroke type. Subsequent research, particularly with hemorrhagic stroke patients, is recommended to further evaluate this hypothesis.

The modest sample size of the current study could potentially impact on the generalizability of our results to the broader stroke population, in particular those cohorts of more severe stroke. However, formal sample size calculations suggest the study was adequately powered, as evidenced by the replication of the significant correlation between DAR and cognition [18]. Moreover, characteristics of participants in the current study were representative of the natural incidence of stroke, including the proportion of ischemic to hemorrhagic strokes [75% ischemic; 5, 37], and the location of cerebral infarction [42% left hemisphere; 32]. EEG data from the stroke group was also compared with previously published normative older adult data [27]. Such comparisons are helpful for describing the observed abnormalities, but cannot explain them, as any number of unmeasured physical, psychological, or environmental factors may have also been contributing to the group differences.

The average MoCA score at 90-day follow-up (21.6) was consistent with previous reports of the magnitude of cognitive impairment following stroke [29]. The prevalence of cognitive impairment in the current cohort (84%) was also in keeping with previous reports in the literature, which have found over three-quarters of stroke survivors exhibit cognitive deficits, including those experiencing a so-called “good” functional recovery [3, 67]. However, participants were not monitored over the time between EEG recordings and cognitive testing. While none of the participants received any form of cognitive remediation from their treating inpatient team, the impact of physical rehabilitation [68–70] or other factors such as the quality of social relationships [71–73] or outpatient care [72] may have influenced cognitive outcomes. Furthermore, seemingly objective instruments such as the MoCA can require some degree of subjectivity, and the methodology in future studies is recommended to include blinding of outcome assessors to reduce the risk of ascertainment bias.

While the MoCA is a standardized instrument validated for use in stroke [4, 29], performance is measured only upon a total score, with no domain-specific scores. Therefore, qEEG sensitivity to deficits in specific cognitive domains (e.g. language, working memory, executive function) remains unknown and can be encouraged as a focus of future investigation. Furthermore, only cognitive outcomes were explored in the current study, and it is unclear if the prognostic value of a single channel EEG device detected herein also applies to functional outcomes. To date, no study (large electrode montage or small) has reported on exactly where the strength of the relationship between neuroanatomical site and functional outcome is greatest, data that would inform a more localised, efficient recording paradigm. Source localization analysis [74] could inform this area of research (e.g. [75]), but is not feasible with the single-channel system employed in the current study.

To our knowledge, this is one of the first studies to apply the NeuroSky Mindset single-channel EEG system in a stroke population. Previous studies have established preliminary validity [26] and reliability [27] of the device. The NeuroSky system is inexpensive compared with lab-based systems, and offers the potential to provide a time and resource efficient, non-specialist alternative to traditional EEG recording systems. EEG expertise remains important, particularly for data analysis. However, new software applications may afford automatized calculation of qEEG indexes described here, and provide a ready prediction of cognitive outcome. The efficiency, affordability, and validity of this novel system deserves consideration in future research and, ultimately, in clinical practice.

In conclusion, early and accurate identification of post-stroke cognitive impairment would represent a major advance in care for the approximately 17 million individuals worldwide who suffer a stroke every year [2]. Acute delta, DAR, and DTR qEEG indexes acquired from a single pre-frontal electrode may enhance early screening for post-stroke cognitive deficits and subsequent cognitive performance after 90-days. Use of the single-channel system in the acute stroke setting was novel and feasible and may leverage clinical decision making for patients with both ischemic and hemorrhagic stroke. Further efforts to evaluate this system in the acute clinical setting and to translate knowledge into viable markers for cognitive outcome are encouraged.
